# American Academy of Dental Science

**Published:** 1900-08

**Authors:** 

**Affiliations:** American Academy of Dental Science


### AMERICAN ACADEMY OF DENTAL SCIENCE.

THE regular monthly meeting of the American Academy of Dental Science was held at Young's Hotel, Boston, Wednesday evening, May 2, 1900, at six o'clock, President Pond in the chair.

The minutes of the previous meeting were read and approved.

A paper was read by Dr. Levi C. Taylor, of Hartford, Conn.; subject, "Sanitary Condition of the Mouth and Teeth, with a Business Method of obtaining the Same."

(For Dr. Taylor's paper, see page 531.)

Also a paper by Dr. Charles A. Porter, of Boston; subject, "Actinomycosis about the Mouth."

(For Dr. Porter's paper, see page 522.)

### DISCUSSION.

*President Pond.*—We are to have a paper to-night by Dr. Levi C. Taylor, subject, "Sanitary Condition of the Mouth and Teeth, with a Business Method of obtaining the Same."

Before presenting Dr. Taylor, who is to read this paper, I think I ought perhaps to give him away, or give away some of the information which he has given me. He admits to-night that he is the sole person who influenced our esteemed fellow-member, Dr. Brackett, to study dentistry.

It gives me great pleasure to present Dr. Levi C. Taylor.

*Dr. Taylor.*—Mr. President and members of the Academy, I want to express to you my gratitude at being present to dine with such a body of men as this. I consider it a very great honor to be invited to come and speak to a society of this character upon a subject of this nature, a subject, probably, of more importance than any other that is brought before the dental profession to-day. There seems to be a great difference in the opinions of men as to our mode of attaining that sanitary condition, and as one of the leading thoughts, I wish to read a very short paper to you this evening.

*Dr. Eames.*—The hygienic condition of the mouth is a subject of great importance; it includes not only the polishing of all surfaces of the teeth, massaging the gums (and I suppose that may include instructions to the patient to do this as far as they are able), and the proper use of antiseptics, but also an inspection of the surrounding soft tissues by the dentist, an examination into the condition of the tonsils and the tongue, about which mucus will lodge and dry. A great many breathe through the mouth, and, of course, not receiving the moisture obtained when the breath enters through the nose, it dries there, forming septic masses in the crypts of the tonsils and other cavities. The effect of this upon the stomach, as well as in the mouth, is deleterious. This occurs by reason of swallowing and by means of the breath.

The means by which a hygienic condition of the mouth is accomplished should be interesting, and I wish that the essayist had gone more into detail as to the methods of sterilizing between the teeth. It seems to me the proper use of nitrate of silver between some of the molar teeth and surfaces which are not exposed to view is a very important aid to sterilizing and keeping them immune from decay. It does seem to me also that an antiseptic spray directed with considerable force from a compressed-air tank will drive out from beneath the margin of the gum, and between the teeth, any accumulations. The use of a brush or scraper over the tongue is something about which we ought to instruct our patients.

*Dr. Brackett.*—I am very glad to acknowledge the dental fathership of the essayist to whom we have listened. We were schoolboys together among the hills of New Hampshire. He is somewhat older than myself, and his influence and success were very strong in leading me to try to learn dentistry. It was in Dr. Taylor's office that I began my dental studies, and the aid and instruction which I there received from him, and from Dr. Riggs also, who was occasionally about the office, have made an impression on me which will never be obliterated. I am always glad to testify my grateful appreciation for what I received from Dr. Taylor.

About the merits of this treatment which he advocates, I do feel very sincerely that the health of the mouth is a thing which should first occupy the dentist's attention in making an examination. I fear there have been in the past, probably more than in the present, practitioners who have construed a search for cavities to be the equivalent of an examination of the mouth. The two expressions should not be synonymous. The investigation of the general conditions of the mouth with reference to all that it presents of suggestion, for the patient's welfare, is one thing. The searching for cavities of decay and finding and caring for them is one element, one part of the whole subject. Some of us who are older have seen in past years a good many instances of people who have been from their childhood once or twice a year for examination and treatment, asking of the dentist only that he render his best service to keep the mouth constantly in good order, and in these mouths there never had been efficient cleansing. There either had been no attention whatever paid to accumulations upon the teeth, or if there had been attention paid, it was to those accumulations which were upon the visible surfaces, and they had been partially removed for the sake of better appearance.

I believe all of our young men who have gone out from our good dental schools in the last quarter of a century have gone out with an appreciation of the desirability of thorough cleansing and the efficient removal of accumulations, wherever they are or however far beneath the margin of the gum, or in whatever out-of-the-way and invisible position, as well as in the prominent and visible places. I think among the patients of the gentlemen who are seated about this board it is the regular thing for a reasonable degree of general health and a reasonable degree of cleanliness to be found; but I feel there is force in that which Dr. Taylor urges, and I believe it is based upon good principles.

One of the latest presentations of what appears to be truth in the etiology of dental caries was made by Dr. J. Leon Williams a few months ago before the New York Odontological Society, and subsequently published in the *Dental Cosmos*. Dr. Williams's paper gave prominence to the idea that the harm of bacterial action comes immediately upon those surfaces of enamel to which the bacteria tenaciously attach themselves and remain. That is, that instead of general conditions of acidity as they exist in the mouth as a consequence of the existence of micro-organisms in the mouth, being the immediate cause of the disintegration of the lime salts of the enamel, it was rather apart from that general acidity. The erosion and disintegration of enamel here were consequent upon direct action produced by firmly and tenaciously adhering patches of micro-organisms. Now, if this is the truth on these points, the practice which our friend has presented appears to meet the indications, at least so far as the mechanics are concerned. Such scouring at short intervals of all enamel surfaces, labial, lingual, and approximal, as shall remove adherent masses of micro-organisms, when supplemented by constant efficient care on the part of the patient, should go far towards the prevention of caries.

Dr. Taylor has told me privately of some cases of his, one case in particular, that of a middle-aged lady, who had usually required each year quite a number of fillings in cavities that were very sensitive. That lady's mouth is one of those to which he has applied this practice, with the result that in a year no cavities have developed, and there has been a subsidence of irritated and sensitive conditions such as had never before been attained.

*Dr. Fillebrown.*—I would like to express my appreciation of Dr. Taylor's effort here to-night. I remember very well receiving a very lasting impression in his office, it must have been twenty-five years ago. I called there one day to meet Dr. Riggs, and the doctor sat me down in his chair and worked over me for about three hours, and made a lasting impression. The impression made me unable to chew with any degree of comfort for a week. But it impressed upon me the importance and efficiency of his treatment.

A great deal is due to Dr. Riggs for developing a scientific practice for Riggs's disease. Dr. Riggs's ideas you will find recorded in Harris's "Principles and Practice of Surgery" years before that. I remember perfectly well doing the same thing myself and of seeing cases that my father treated years before I heard of Dr. Riggs; and still neither my father nor myself had the farsightedness to formulate it and bring it to the attention of the profession in the form that Dr. Riggs did. I am exceedingly sorry that Dr. Riggs had not a little more of the literary turn, and that he should not have put his ideas in the form of a paper and had it go on record, so that there should be no mistake and no question as to his claims to originality in the matter of developing the treatment.

I long have had patients who have done themselves as well as myself the favor to come in for every month at least, or oftener if any trouble occurs. What I say to my patients is, "If these foreign substances are not kept off the teeth the teeth surely will go to ruin, and whenever you see the gum thickening a little, or growing a little red, you may know there is trouble and you need to have treatment." Certainly teeth ought to be attended to once a month. I have in mind one patient who calls every two, four, or eight weeks, and has done so this eight or ten years. When this patient first came to me the teeth were loose and very near destruction. To-day they are firm and healthy.

I remember as long as twenty years ago or more a patient came to me with teeth standing so they looked like a Virginia fence, leaning out and in, and by the judicious use of scalers and by polishing the teeth they gradually came back into place and presented a very creditable appearance indeed.

The plan that has been described here by Dr. Taylor to-night is unquestionably the right thing. If teeth can be kept absolutely clean there is no doubt but that caries can be prevented. I am glad he is so fortunate as to have so many of his patients follow him. I wish the whole community could be of that kind, but the fact of it is, they are not and cannot be. It is very desirable that a plan so perfect and well developed should appear, that those who are willing to be saved may be.

*Dr. Bradley.*—There is one suggestion that Dr. Taylor gave us, about the younger dentists feeling an ambition to work in gold and acting as though the cleansing of teeth and keeping the mouth in a hygienic condition was beneath their ambition, which I wish to say a few words about.

I remember some years ago seeing a young man, a graduate of a dental school, who presented himself to take a further post-graduate course, and there was given to him a patient, what he termed "a mere cleaning case," and he said to me, "I have taken one degree, and I know how to clean teeth. What I want to do is to fill teeth." He wanted to spend his time in filling teeth, and so on. But after some conversation, in which I in a pleasant way showed him the importance of it, he came to the conclusion that there was something to be done in cleaning teeth and getting the mouth into a satisfactory condition. Before the young man left Boston he acknowledged to some of us that he had found the necessity of doing this work in a thorough manner, not simply for the personal appearance, but that the teeth might be really clean.

There is so much cleaning done just to have the teeth look well on the surface, and not to make them clean and healthy, that I think the importance of it cannot be over-estimated in getting at the difficult places. I question whether many of us will get to see our patients once a month, but I am very sure that the more perfectly we can get our patients to keep their teeth, with our assistance, the less decay we shall have, and it is the field in which we must work, the prevention of decay, not the restoration of the part after the damage is done, but trying to prevent it as much as possible; and so to that extent I am in hearty sympathy with the sentiments of the essayist this evening.

*Dr. Williams.*—Dr. Taylor refers to massage as one part of the treatment he recommends. I would like to ask what was the operation of the massage in his treatment?

*Dr. Taylor.*—This gentleman asks the question, what I mean by massaging. To massage is to rub, as I understand it, and stimulate by friction a healthy action in any part of the body. I was talking with a physician in New York City a few days ago about this matter of massaging teeth. He said, "You may massage any part of the body, and that part that you do massage will improve materially in advance of all the other parts, and I do not know why the teeth are not susceptible to the same results."

The gentleman at the other end of the table thinks it would have been interesting if I had gone a little more into detail. But coming before a body of men of this character, it is a very difficult matter to put in black and white all the little details; it almost looks as though we were questioning their ability, and for that reason I touched as lightly as I thought advisable.

The matter of system, making yearly contracts with the patients, is simply to have them pay for something, and then they feel that they must come,—not to make a contract in the sense of the word that is so objectionable in medicine, but when a patient pays for a thing he is pretty sure to come and get it. It is the only way that you can compel them to come each month regularly, systematically, and on time; and when you do that you will get them.

I want to see those patients often enough to massage the teeth with the orange-wood stick and pumice and do all my work with that after the mouth is under complete subjection. I will say this, that it will take, in ordinary mouths, from four to eight months to get the mouth under subjection. Now, that may seem a little strange to you. If you will take a case and do the very best you can, cleaning every part thoroughly, spending hours, and then follow it month by month for six months, and note the improvement that you will get, you will be elated; it will please you. It is something that has surprised me. I had no idea that the constant systematic work would produce the advanced condition of pleasure that it produces to see how the mouth improves.

These cases that I referred to in my paper as being two of the worst were not cases of Riggs's disease proper, nor of pyorrhœa; they were cases where the coating would come around the neck, and the teeth were diseased with what we call the white chalky decay. You have seen that many times around the necks of the bicuspids, very rapid decay, very sensitive, very painful to the touch. Gentlemen, if you will polish and, as I call it, "massage" (call it what you please) often enough, you will be able to nearly or quite prevent decay. In extreme cases of this kind you had better take them every two weeks for a few months. I want to say right here that I have now a patient that has been under my care for five months, the worst case of sensitiveness on the cuspids that I ever saw. She is a poor girl. I have had interest enough in the case to say to her, "If you will come every two weeks I will do my best for you free of charge; I want to know what I can do." I will say this, that I saw her for four months before I could even touch the teeth with the stick and pumice without her just getting wild. I am getting now so that it is coming into shape and I can begin to rub them very considerably. It is painful yet, but I predict that in about two or three months more I will succeed in reducing the pain. I have tried tannic acid and an innumerable number of remedies, and not one of them seemed to have any effect. I have followed this four months, and now am just beginning to see some results. Am not yet through with the case.

Dr. Fillebrown refers to my having fifteen patients. Gentlemen, there is not a man in this room who cannot have within three years' time three times fifteen patients who will stand by him straight and square if he handles his patients rightly. These patients are the most appreciative that I have ever had. The results are wonderful.

As regards what he says about Dr. Riggs's treatment, his case I remember very well, some twenty-five years ago; it was an advanced case of pyorrhœa, trouble on the lower teeth; his upper teeth were not so much affected, if I remember rightly. He treated the under teeth. I am not speaking of treating the advanced cases. I want you to take this method up in your common, everyday practice, and study each case well.

It is not an uncommon thing to see mouths that have been cleaned as I had one a short time ago. A man came in and said to me, "Doctor, I have come to you because I want better work than I have been getting." I told him his teeth needed cleaning. "Why, I had them cleaned only a week or two ago," he said. "My dear sir, you haven't had your teeth cleaned in ten years." "Well," he said, "if I haven't, I want them cleaned." It was not a case of pyorrhœa at all; it was a simple case of cleaning teeth. It took me one hour and twenty minutes to finish up, after the other man had spent five or ten minutes with his engine. There was no pyorrhœa.

I want to speak of one particular case that I have followed for twenty-five years. A lady had a front under tooth where the gum had receded the full length of the tooth almost to the apex. The apex was not exposed. She commenced with Dr. Riggs twenty-five years ago, at the time our friend was there. He treated it as long as he lived. Afterwards it came into my hands. I have followed that case once in two or three months ever since, and I am not able to see that there is one bit of progress in the disease working down. Almost every time that she presents herself there is quite an amount of the white soft tartar, but this can be almost entirely removed with the stick and pumice without an instrument. But what I want is to get at this often enough so the stick and pumice will do the whole business, and do it well. But it never will be done thoroughly with the engine because it is so deceitful. You will do what is visible to the eye, but you do not get up under the gum. A man says to me, "We could retouch it afterwards." Yes, but you do not do it. You have cleaned off the face of the tooth; you have cleaned it beautifully, and what will be the result? You won't take your stick and work up under the gum, because you haven't your guide to go by. If you do what is under the gum first with your stick and pumice, and do it well, when you are through there will be nothing left to be done with the engine.

I am well aware that in spending the time that I believe in spending it is necessary that we get a higher fee than most men are inclined to charge for such work; and that is one of the beauties of the yearly contract system, where you guarantee a mouth against decay. It is not guaranteeing against breakage,—it is not guaranteeing against a pulp that may die under a large filling that has been in the mouth fifteen to thirty years,—but it is against actual decay. It works both ways. The knife is a double-edged one. If you do not do your work well you will have teeth to fill.

As regards the fees, that is a very difficult question, and I cannot draw any line for all classes of men, because the high-priced man of the city cannot afford to work for the price of the cheaper country practitioner, and it would not be right that he should be asked to. But the basis upon which to work would be to ascertain so far as you can what you would obtain from that patient under the ordinary methods of treatment annually, not taking it some year when they have neglected it for three or four years, but what you average to receive. Make this a basis, then add twenty-five to thirty per cent., and you will not be far out of the way in making your yearly contract. You can very easily make your own estimates after you stop to think and figure along this line. This is a question that can be regulated by all of us.

I want every one to feel that they must do their work faithfully. Many first-class practitioners, so considered by the masses and by the profession, are not doing this particular work well. They are doing beautiful filling, but it would be far better for them and far better for their patients to prevent the necessity for filling. It can be done.

Subject passed.

*President Pond.*—You all remember a short time ago we had a very interesting meeting, made so quite largely by the efforts of Dr. Porter. Dr. Porter has kindly consented to come again and give us another paper, and I have no doubt will add another very interesting meeting to close our series for this winter.

I have now the pleasure of presenting Dr. C. A. Porter, who will give us a paper on actinomycosis about the mouth and a report of ten cases.

DISCUSSION.

*Dr. Briggs.*—This is a very interesting paper to us, perhaps more so than we realize. As I look back a number of years I remember cases that it seems to me may have been due to actinomycosis.

I recall one case in particular that was suggested to me by Dr. Porter's treatment, from the fact that after working on this case a good deal I finally put the patient on iodide of potash, and he made better progress than he had under my surgery.

Last winter Dr. Hardy called me in consultation for a young school-girl; an examination showed a large abscess at the side of the jaw, about opposite the second molar. It was almost ready to break through the skin, and the anxiety was to prevent its breaking through and making a scar on the young woman's face. The second molar had a history of having an abscess on it, for which it had been treated. And through that tooth I tried to reach the fistula and the abscess. We only found one canal, and the case not yielding at all, and the patient being in a very low state, weak and sick, the tooth was finally extracted in order to get at the abscess better. The tooth proved to be a single-rooted tooth.

The abscess was opened, but did not yield to treatment, and the danger grew more imminent that it would break through the face. Dr. Munroe was called in consultation, and at his suggestion a specimen was sent for microscopic examination, which proved it to be a case of actinomycosis, with the subsequent treatment and cure as described by Dr. Porter. This is a case in practice which shows that it does occur, and I have no doubt that many other cases that I can recall were such.

*Dr. Fillebrown.*—I want personally to thank Dr. Porter for bringing these cases before us here. They are extremely instructive, and while I haven't anything to say to-night other than this, I hope to make further use of it in the future.

*Dr. Porter.*—I did not intend to have you understand that I really think that this disease is a common one. When any one has a hobby, they interpret things in the light of that hobby, as I said before, and I began to think that actinomycosis must be a common thing. I do not doubt that carious teeth and roots are the cause of the great majority of alveolar abscesses; but I think it is interesting to have in mind the fact that a diagnosis can be made, and actinomycosis can be ruled out by a microscopic examination. Undoubtedly in certain cases it would be found that they were due to actinomycosis, and the surgeon would manage that case differently from what he would an ordinary abscess. I really think that thorough removal would give the patient less scars than if the disease progressed. Resort first to thorough curetting, and then if the disease returns, tell the patient that an incision would give him less trouble than simply curetting three, four, or five times.

*Dr. Bradley.*—I am sure that we all appreciate the opportunity of listening to two such papers as we have this evening, and I shall only voice the expression of the fellows of the Academy in moving that the appreciation be shown by a vote of thanks both to Dr. Taylor, of Harvard, and to Dr. Porter, who has come in to give us the result of his work. I move that we pass them a vote of thanks for our appreciation.

Charles H. Taft,
*Editor American Academy of Dental Science.*

